# Predictors of preventive behavior of nosocomial infections in nursing staff: a structural equation model based on the social cognitive theory

**DOI:** 10.1186/s12913-021-07205-6

**Published:** 2021-10-31

**Authors:** Seyed-Mousa Mahdizadeh, Seyedeh Belin Tavakoly Sany, Davood Robat Sarpooshi, Alireza Jafari, Mehrsadat Mahdizadeh

**Affiliations:** 1 Department of Medical-Surgical Nursing, School of Nursing and Midwifery, Mashhad University of Medical Sciences, Mashhad, Iran; 2 Nursing and Midwifery Care Research Center, Mashhad University of Medical Sciences, Mashhad, Iran; 3 Department of Health Education and Health Promotion, Faculty of Health, Mashhad University of Medical Sciences, Mashhad, Iran; 4 Social Determinants of Health Research Center, Mashhad University of Medical Sciences, Mashhad, Iran; 5 School of health, Sabzevar University of Medical Sciences, Sbzevar, Iran; 6 Department of Health Education and Health Promotion, School of Health, Social Development and Health Promotion Research Center, Gonabad University of Medical Sciences, Gonabad, Iran

**Keywords:** Nosocomial infections, Hospital, Nurse, Social cognitive theory, Structural equation modeling

## Abstract

**Background:**

The occurrence of nosocomial infections remains a health threat to patients and hospital staff. This study applied social-cognitive theory for predicting determinants of nosocomial infections control behaviors in hospital nursing Staff.

**Methods:**

In this cross-sectional study, 280 nurses and assistant nurses were selected by random sampling from intensive care wards including CCU, ICU, NICU, dialysis of educational hospitals in Mashhad, Iran in 2020. Data were collected using a 5-point Likert scale structural questionnaire based on social cognitive theory constructs. Using the structural equation modeling method, direct and indirect relationships of social cognitive factors on preventive behaviors of nosocomial infections were analyzed via AMOS 23.0.

**Results:**

Our results showed that self-regulation, outcome expectations, and barrier constructs had a direct effect on behavior and the highest effect was related to self-regulation structure (*p* < 0.001). The constructs of social support, modeling, perceived environment and Task self-efficacy had an indirect effect on behavior and the most impact was related to the constructs of perceived environment (*p* < 0.05).

**Conclusion:**

Considering that self-regulation, outcome expectation and barriers have a significant effect on following the preventive behaviors of nosocomial infections in nursing staff. It is suggested that policymakers and planners try to reduce barriers, strengthen behavioral motivation, and empower nursing staff by teaching self-regulatory strategies.

## Background

Although the quality of health services has been improved and infection prevention and control methods have been developed, the occurrence of nosocomial infections remains a health threat to patients and hospital staff [1]. Due to the biological characteristics of nosocomial infection pathogens, such as antibiotic resistance and high pathogenicity, as well as the sensitivity and frailty of hospitalized patients, there is a strong relationship between nosocomial infection and mortality [2].

Nosocomial infections affect many patients around the world. Roughly, 15% of hospitalized patients suffer from these infections. The economic losses from these infections are increasing. The incidence rate in developed countries is about 3.5 to 12%. The prevalence of these infections in underdeveloped countries is about three times higher than in developed countries [3]. In these countries, this rate varies between 5.7–19.1% [4]. In Iran, most of these infections occur in intensive care units and surgical wards [5]. Giving the role and duties of clinical staff in hospitals, they can be a factor in the transmission of nosocomial infections. Adherence to infection control instructions such as the use of masks and cleaning and disinfection of hands and equipment after contact with patients and their contaminated equipment is the most important duties of these employees. Therefore, adherence to these behaviors can reduce nosocomial infections [3].

To prevent and control nosocomial infections, it is essential to determine the predictors of preventive behaviors of nosocomial infection. For this end, effective models are needed to improve patient safety and reduce nosocomial infections [6]. Social cognitive theory is a comprehensive theory. According to this theory, behavior is formed from the interaction of environmental, personal and behavioral factors [7]. Self-regulation, self-efficacy, outcome expectation, and environment are important constructs of social cognitive theory [8, 9]. These constructs can be used as a guide for developing procedures and implementing of interventions to change healthy behaviors. Bandura emphasized that self-regulation strategies (i.e., ability to self-monitor and evaluate their behavior) must be used to set goals and plan to adopt and maintain a behavior [8]. Self-monitoring is a guide to evaluating one’s progress toward goals, which leads one to a behavior [10]. The results of the study indicated that self-regulation is one of the determinants of behavior related to standard precautions such as hand hygiene [11].

A study showed that nurses in the intensive care unit need to strengthen self-efficacy as one of the internal factors in order to carry out infection control-related behaviors [12]. Another study also showed that self-efficacy is related to nursing care [13].

In previous studies, the effect of social cognitive factors on hand hygiene as one of the measures in the control and prevention of nosocomial infections has been investigated, but the effect of social cognitive factors on compliance with standard precautions for nosocomial infection control is not well understood. In a study that was conducted to identify social cognitive factors affecting on hand hygiene in hospital nurses, subjective norms, attitudes, perceived behavioral control, risk perception and intention were identified as important predictors of hand hygiene [14]. Structural equation modeling helps researchers determine how the theory works to influence the outcome because it approaches the model through predictor variables, the mediation, and the consequence of the variables’ relationships with each other [15]. It is used for testing several theoretical models, which define the structure of constructs and their relationship with each other [16–19].

This study aimed to provide Structural equation model to predict social cognitive theory on behaviors related to prevent and control of nosocomial infections in nursing staff.

## Methods

### Study design, setting and participant requirement

This study used a cross-sectional, self-reported design in evaluating the predictors of behaviors related to prevent and control nosocomial infections among nursing staff. Data were collected from August to December 2020. A convenience sample of 280 nursing staff were selected for the study. The research sample was selected from the nursing staff (nurse and assistant nurses) working in the intensive care units including CCU, ICU, NICU, dialysis units in academic hospitals in Mashhad, Iran. Inclusion criteria were willingness to participate in the study, employment in intensive care units for more than 6 months. Exclusion criteria was incomplete of the questionnaire by study participants.

### Questionnaire

This study used a self-report questionnaire to collect data. This questionnaire included 2 part; part 1 included demographic characteristics (e.g., age, gender, educational status, training about nosocomial infections and job). Part 2 elicited constructs of social cognitive theory including perceived barriers, task self-efficacy, self-regulation, outcome expectations, perceived environment, modeling, social support, and behaviors related to prevent and control of nosocomial infections. This part of questionnaire included 36 items (perceived barriers 5 items, task self-efficacy 4 items, self-regulation 5 items, outcome expectations 4 items, perceived environment 2 items, modeling 3 items, social support 3 items, and practices of nosocomial infection control 10 items). Each item in this section were scored from 1 to 5 on a Likert scale from completely disagreement to completely agreement.

The content validity methods were used to assess the validity of the questionnaire. For this purpose, the opinions of 10 experts in health education, nursing, and infection control on the items of the questionnaire were received and the items were modified or deleted based on their suggestions. Content Validity Index (CVI) and Content Validity ratio (CVR) assessed for each items of structures (self-efficacy, modeling, social support, self-regulation, perceived environment, perceived barriers, outcome expectation, and behaviors). The CVR value for the items was between 0.76 and 0.91. Also, the CVI value for the items was between 0.81 and 0.98. The reliability of the instrument was evaluated using test-retest method and Cronbach’s alpha test. For external reliability, 30 nurses and assistant nurses completed the questionnaires twice with an interval of 10 days, except for the study sample, and the data were analyzed by Pearson correlation coefficient. Cronbach’s alpha test was also used to assess internal validity (Table 1).

**Table 1:** The results of reliability tool based for the constructs of social cognitive theory

Subscale	Cronbach’s alpha	Test-re test
Perceived barriers	0.832	0,810
Perceived environment	0.628	0,870
Self-regulation	0.859	0,892
Outcome expectations	0.842	0,861
Self-efficacy	0.830	0,924
Social support	0.803	0,893
Modeling	0.730	0.792
Behavior	0.933	0,911
All constructs	0.816	0.781

### Statistical analyses

**Fig. 1:**
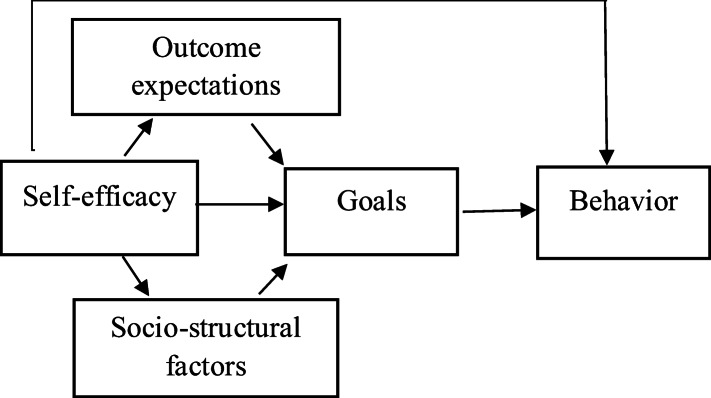
Theoretical model of Social cognitive theory

The collected data were analyzed using IBM SPSS Statistics 21.0 (IBM Corp., Armonk, NY) for evaluating the normality of variables and descripting of statistics. In this study, the principles of social cognitive theory were used to structural equation modeling. The basic conceptual model of behavior predictors based on social cognitive theory [20], which provides a theoretical framework for our study, is illustrated in Fig. 1. The fit of the constructs of social cognitive theory for behaviors related to the control of nosocomial infections in hospital nursing staff was examined using the latent variable structural equation model (SEM) with AMOS software version 24.

Structural equation modeling is a detailed statistical method for testing models that contain both causal relationship and correlations between observed variable and latent variables. SEM is used in social and behavioral sciences, education, biology, economy, marketing and medicine, which is based on substantial or suggested theories that describe and explain phenomena under investigation. We used R2 and the path coefficients to test the fit of the model. Before carrying out the SEM analysis, the normality of variables was examined [21]. All the indicators in the model were treated as reflective indicators of their respective constructs. We developed the model (theoretically driven) with eight latent factors. The modification index suggested correlation of several error terms. After covering the errors, we obtained the acceptable model fit. The goodness-of-fit of the model was confirmed using the χ2 statistic, RMSEA (Root-Mean-Square Error of Approximation), CFI (Comparative Fit index), IFI (Incremental Fit Index), PNFI (Parsimonious Normal Fit Index), and PGFI (Parsimonious Good Fit Index) [15]. Parameter estimation and effect analysis were performed using the bootstrapping method. Statistical significance was set at *P* < 0.05.

### Ethics approval and consent to participate

This study was conducted in accordance with the principles of Declaration of Helsinki. All methods were carried out in accordance with relevant regulations and guidelines. This study is based on a research study approved by the ethics committee of Mashhad University of Medical Sciences with the ethical code IR.MUMS.REC.1398.005. Informed written consent was obtained from all participants before start of this study. By completing consent form, participants were informed about the purpose and method of the study. Participants were also informed that the researchers are committed to answering their questions and that their information was kept confidential. In addition, participants were aware that their participation in the study was voluntary and that they could leave the study at any time.

## Results

Participants had a mean of age 35.42 ± 8.06 years. Fifty-six percent of participants in this sample were men, and most of the samples had a bachelor’s degree. Eighty percent of the samples participated in an infection control-training course (Table 2).

**Table 2:** General Characteristics of Participants

Variables		N	%
Gender	Male	157	56.1
	Female	123	43.9
Educational status	Diploma and lower	84	29.6
	Bachelor’s degree	181	65.0
	Master’s degree and higher	15	5.4
Infection control training course	Yes	223	80.3
	No	57	19.7
Job	Assistant nurse	149	51.48
	Nurse	131	48.52

Before performing the structural equation model, first, the remote points and the normality of the variables were examined. The remote points were checked with the help of the value of mahalanobis distance of each observation and if necessary, they were removed. In the study of critical ratios of skewness or elongation of variables, the normality of multivariate was confirmed, therefore, the maximum likelihood method was used to estimate the parameters. Then, the structural equation model of social cognitive theory constructs was examined and analyzed. Based on the results of the indicators, the desired model has been accepted) X^2^ = 1184.739 (*p* > 0.05), df = 572, X^2^/df = 2.07, CFI = 0.910, RMSEA = 0.062, PNFI = 0.739, and PGFI = 0.811, IFI = 0.910, *p* < 0.001).

The total effect size of social support on perceived environment was 0.502, perceived environment on task self-efficacy 0.264, social support on task self-efficacy 0.550, outcome expectations on self-regulation 0.729, task self-efficacy on self-regulation 0.140, perceived environment on self-regulation 0.037, self-regulation and outcome expectations and barrier on behavior Respectively 0.490/0, 0.780, − 0.211. Most of the direct and indirect causal effects were statistically significant and positive. Most of the direct and indirect causal effects were statistically positive and significant (Table 3).

**Table 3:** Direct and indirect effects of constructs social cognitive theory

Predictors	Causal Effect		
	Direct	Indirect	Total effects
Social support → Environment	0.502*	–	0.502
Social support → Modeling	0.686*	–	0.686
Environment Perception → Task self-efficacy	0.264*	–	0.264
Social support → Task self-efficacy	0.417*	0.133**	0.550
Modeling → Outcome expectations	0.563*	–	0.563
Environment Perception → Barrier	−0.980*	–	−0.980
Outcome expectations → Self-regulation	0.729*	–	0.729
Social support → Self-regulation	0.129**	0.358**	0.487
Task self-efficacy → Self-regulation	0.140**	–	0.140
Social support → Outcome expectations	–	0.386**	0.386
Social support → Barrier	–	−0.492**	−0.492
Modeling → Self-regulation	–	0.411**	0.411
Environment Perception → Self-regulation	–	0.037**	0.037
Self-regulation → Behavior	0.490*	–	0.490
Outcome expectations → Behavior	0.423**	0.357**	0.780
Barrier → Behavior	−0.211**	–	−0.211
Social support → Behavior	–	0.179**	0.179
Modeling → Behavior	–	−0.037	−0.037
Environment Perception → Behavior	–	0.225**	0.225
Task self-efficacy → Behavior	–	0.068**	0.068
Through total causal effect	2.729	1.625	4.354
Percentage of direct and indirect effects	2.729/4.354 =62.68%	1.625/4.354 =37.32%	

*P < 0.001, **P < 0.05

**Fig. 2:**
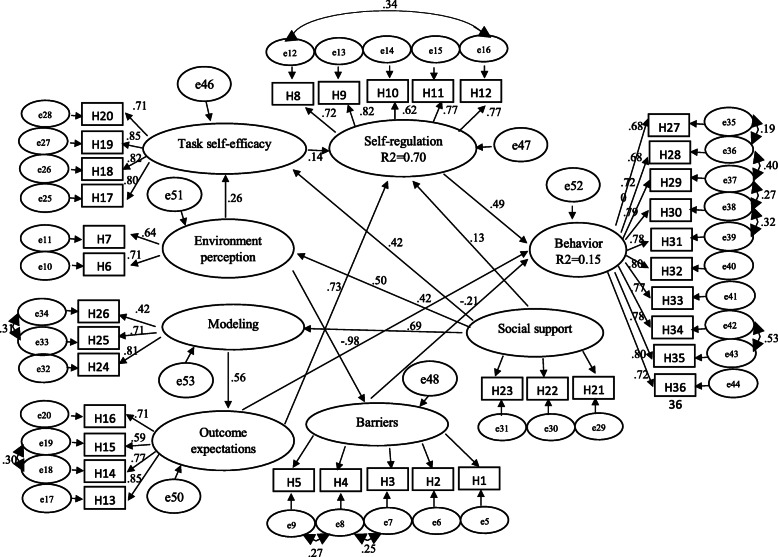
SEM and Path coefficient between constructs of social cognitive theory and preventive behaviors of nosocomial infections (R2: Squared Multiple Correlation)

The constructs of barriers, perceived environment, outcome expectations, task self-efficacy, social support, and modeling were able to predict 70% of the variance of Self-regulation construct. The variance of the structures of this model in predicting the variance of the structure of behavior are shown in Fig. 2.

The results of the structural equation model showed that self-regulation, outcome expectations, and barrier constructs had a direct effect on behavior and the highest effect was related to self-regulation structure. Also, Percentage of total direct effects of structures are greater than the total indirect effects of structures. The structures of social support, modeling, perceived environment and task self-efficacy had an indirect effect on behavior and the greatest effect was related to perceived environmental structures (Table 3).

## Discussion

In this study, the role of social cognitive theory constructs as predictive factors of behavior related to the control and prevention of nosocomial infections in hospital nursing staff was evaluated. Structural equation analysis shows that the theoretical model is suitable for the data and explains the nosocomial infections control behavior in the hospital nursing staff. In the test of goodness-of-fit of the final modified model, the absolute fit indices (χ2, and RMSEA), the comparative fit indices (IFI and CFI) and parsimonious fit indices (PCFI and PNFI) met the criteria, indicating that the model-fit well with the data. The literature described that a good model is with insignificant χ2 (*p* ≥ 0.05), IFI ≥ 0.90, PGFI ≥0.50, RMSEA ≤0.08, PNFI ≥0.50, and CFI ≥ 0.90 [22–24].

Our research results show that self-regulation directly plays an important role in the performance of hospital nursing in controlling and preventing nosocomial infections. Consistent with these results, a study showed that the process of self-regulatory is an important predictor of hand hygiene [25]. In fact, when individuals control their performance, they are more likely to set more realistic and challenging goals, thereby helping to overcome behavioral barriers [26].

In this study, the results show that independent of self-regulatory behaviors, self-efficacy has little effect on behaviors. Consistent with our results, studies have shown that one of the determinants of healthy behavior is self-efficacy, which can predict various healthy behaviors, including prevention of nosocomial infections and hand hygiene [27, 28]. Although self-efficacy is often a powerful predictor of behavioral adoption and maintenance in healthy behaviors, a meta-analysis showed that self-efficacy has different effects on healthy behaviors are [29]. Researchers also describe people who have a high level of self-efficacy trying harder to achieve their goals when faced with obstacles [30].

In the present study, outcome expectation is influenced by modeling, and directly and indirectly affects the behavior of nursing staff through self-regulation. Other researchers have also shown that observational learning can enhance outcome expectations and can influence behavior [31]. Contrary to these results, in another study that showed the results of predicting the constructs of protection motivation theory integrated with the outcome expectation construct of the social cognitive theory related to protective measures against Ebola infection, the outcome expectation construct did not predict the protective behaviors of nurses against Ebola infection [32].

The results of this study indicate that the perceived environment affects barriers. Barriers also have a direct and negative effect on the preventive behavior of nosocomial infections in hospital nurses. Consistent with these results, other studies have shown that barriers such as insufficient knowledge, personal attitude and judgment, environmental constraints, and insufficient leadership skills have a negative effect on nurses’ adherence to standard precautions [33–36].

In this study, social support is another determinant of nosocomial infections prevention behavior, which indirectly affects it. The influence of social support on preventive behaviors of nosocomial infections was through self-regulation, self-efficacy, perceived environment and modeling. Based on these results, the literature describes that self-efficacy and social support are important facilitator of self-regulation, especially planning [37]. In another studies, social support, including institutional and management support for stablishing a safe environment and providing facilities, had a significant impact on compliance with standard precautions (such as hand hygiene) [38–40].

Our results showed that modeling has a negative effect on the preventive behavior of nosocomial infections in hospital nursing staff. This factor also had an indirect effect on behavior through barriers. The literature points out that the role of a mentors as a role model affects the hand hygiene behavior of nursing students [41]. In another study, the lack of appropriate role models was identified as one of the problems of non-compliance with standard perceptive measures, which was caused by environmental factors and barriers (including conflicts between professional, unsupported organizational culture and financial issues [42].

In the present study, perceived environment had an indirect effect on the preventive behavior of nosocomial infections in the hospital nursing staff through barriers. Consistent with these results, earlier studies have shown that environment factors (for example, lake of access to personal protective equipment, heavy workload, and crowded ward) can have a significant impact on compliance with standard precautions by healthcare staff [43, 44].

### Limitations and future research

This study tested the predictive effect of social cognitive theory constructs on a set of behaviors preventing nosocomial infections. While, previous studies have examined factors related to hand hygiene behavior. This study was conducted in all intensive care units in several hospitals, therefore, the generalized findings are appropriate. One of the weaknesses of the study is that the effect of knowledge and demographic factors such as age, gender and education, which have been identified in previous studies as important factors on adherence to preventive behaviors of nosocomial infections, was not examined. This study was also conducted in teaching hospitals that have special conditions in the hospitalization, treatment and nursing-care processes, and therefore it is necessary to examine the interaction of environmental, personal and behavioral factors in other non-teaching and private hospitals.

## Conclusion

The present study demonstrates that self-regulation, outcome expectations, and barriers have a significant impact on adherence to preventive behavior of nosocomial infections in nursing staff. In addition, current analyzes shows that although self-efficacy is an important precursor to self-regulation, social support was even a stronger predictor. The social support indirectly affected self-regulation through self-efficacy. In addition, social support directly led participants to use self-regulation strategies, and therefore, more compliance with prevention behaviors of nosocomial infections. It is suggested that policy makers and planners try to reduce barriers, strengthen behavioral motivation, and empower nursing staff by teaching self-regulatory strategies such as goal setting, self-monitoring, and planning to promote behaviors that prevent nosocomial infections. In addition, it is recommended that future behavioral psychology models be used in future studies to test the relationship between other variables and preventive behavior of nosocomial infections in other health care providers and non-governmental hospitals.

## Data Availability

Data and materials can be requested from the corresponding author.
